# Protocol for the Adaptation of a Direct Observational Measure of Parent-Child Interaction for Use With 7–8-Year-Old Children

**DOI:** 10.3389/fpsyg.2020.619336

**Published:** 2021-01-14

**Authors:** Shannon K. Bennetts, Jasmine Love, Elizabeth M. Westrupp, Naomi J. Hackworth, Fiona K. Mensah, Jan M. Nicholson, Penny Levickis

**Affiliations:** ^1^Judith Lumley Centre, La Trobe University, Melbourne, VIC, Australia; ^2^Murdoch Children’s Research Institute, Melbourne, VIC, Australia; ^3^School of Psychology, Deakin University, Melbourne, VIC, Australia; ^4^Parenting Research Centre, Melbourne, VIC, Australia; ^5^Department of Paediatrics, The University of Melbourne, Melbourne, VIC, Australia; ^6^Melbourne Graduate School of Education, The University of Melbourne, Melbourne, VIC, Australia

**Keywords:** parent-child interaction, observation, measurement, sensitive responding, positive mutuality, parent responsiveness, parent sensitivity

## Abstract

**Objective:**

Parenting sensitivity and mutual parent-child attunement are key features of environments that support children’s learning and development. To-date, observational measures of these constructs have focused on children aged 2–6 years and are less relevant to the more sophisticated developmental skills of children aged 7–8 years, despite parenting being equally important at these ages. We undertook a rigorous process to adapt an existing observational measure for 7–8-year-old children and their parents. This paper aimed to: (i) describe a protocol for adapting an existing framework for rating parent-child interactions, (ii) determine variations in parents’ sensitive responding and parent-child mutual attunement (‘positive mutuality’) by family demographics, and (iii) evaluate the psychometric properties of the newly developed measure (i.e., inter-rater reliability, construct validity).

**Method:**

Parent-child dyads completed one home visit, including a free-play observation and parent questionnaire. Dyads were provided with three toy sets: LEGO^®^ Classic Box, Classic Jenga^®^, and animal cards. The *Coding of Attachment-Related Parenting (CARP)* was adapted for use with 7–8-year-old children, and rating procedures were streamlined for reliable use by non-clinician/student raters, producing the *SCARP:7–8 Years.* Trained staff rated video-recorded observations on 11 behaviors across two domains (five for parents’ sensitive responding, six for parent-child positive mutuality).

**Results:**

Data were available for 596 dyads. Consistently strong inter-rater agreement on the 11 observed behaviors was achieved across the 10-week rating period (average: 87.6%, range: 71.7% to 96.7%). Average ICCs were 0.77 for sensitive responding and 0.84 for positive mutuality. These domains were found to be related but distinct constructs (*r* = 0.49, *p* < 0.001). For both domains, average ratings were strongly associated with the main toy used during the observation (*p* < 0.001, highest: cards, lowest: LEGO^®^). Adjusted multivariate linear regression models (accounting for toy choice) revealed that less sensitive responding was associated with younger parent (*p* = 0.04), male parent (*p* = 0.03), non-English speaking background (*p* = 0.04), and greater neighborhood disadvantage (*p* = 0.02). Construct validity was demonstrated using six parent-reported psychosocial and parenting measures.

**Conclusion:**

The *SCARP: 7–8* Years shows promise as a reliable and valid measure of parent-child interaction in the early school years. Toy selection for direct observation should be considered carefully in research and practice settings.

## Introduction

Early childhood (age 0–8 years: World Health Organization, 2020) is recognized as a critical period of growth and development, shaped by interactions between biological, cultural and societal factors. Considerable emphasis has been given to the earlier years of this period, with less focus on the later years of early childhood (7–8 years) during which children transition to school and are required to adapt to a formal learning environment (Bardack et al., 2017). Although children are increasingly exposed to influences beyond their primary caregivers (e.g., peers, teachers), parents remain an integral part of their children’s learning and development in the early school years (Boldt et al., 2016), providing opportunities for children to develop school readiness, such as behavior and emotion regulation, attention, and social skills (Morrison et al., 2003; Bardack et al., 2017). When the parent-child relationship is based on secure attachment and features sensitive parenting behaviors, children are more likely to reach their academic potential, and to develop better self-regulation and social skills (e.g., Morrison et al., 2003; Keown, 2012). However, there remains a lack of brief, robust observational tools to assess parent-child interaction in the early school years, particularly for use by non-clinicians. Drawing on data from a large cohort of Australian parent-child dyads at child age 7–8 years, we describe and evaluate an adapted direct observational measure of parent-child interaction.

Parenting behaviors, such as sensitively responding to a child’s needs, make an important contribution to child development from infancy (Bornstein and Tamis-LeMonda, 1989). Although the time children spend with their parents decreases at school entry, quality parent-child interaction, specifically interaction that supports children’s changing developmental needs, continues to contribute to children’s socioemotional and behavioral development (Iarocci and Gardiner, 2015). Sensitive responding is a concept related to attachment theory (Bowlby, 1997), whereby parents recognize and respond promptly and appropriately to their child’s cues, offering guidance, accepting and encouraging their child’s autonomy, and demonstrating warmth toward their child (Matias, 2006). Such parenting behaviors are linked to a range of positive child outcomes, such as socioemotional development (Scherer et al., 2019) and behavioral regulation (Moss et al., 1998), which are essential for optimizing learning opportunities at school (Williams and Berthelsen, 2017). In addition to parental behaviors, it is important to consider the transactional nature of interaction, whereby parents and children recognize and respond to each other’s verbal and non-verbal cues. ‘Positive mutuality’ or ‘synchrony’ captures the extent to which parents and children are ‘in-tune’ and mutually responsive (Matias, 2006). Greater positive mutuality has been linked to less anti-social behavior and inattentiveness, better social skills, and better behavioral regulation (Criss et al., 2003; Deater-Deckard et al., 2004; Keown, 2012; Hedenbro and Rydelius, 2019).

Measurement of any behavior is subject to bias (Bland and Altman, 1999; Bennetts et al., 2016, 2017b) and both parent-report and direct observational measures of parent-child interaction contribute uniquely to the research evidence (Aspland and Gardner, 2003; Wysocki, 2014). For example, while parents can reflect on behaviors over a longer period of time, parent-reported measures can be subject to socially desirable responding or a lack of objective awareness about interactions (Funamoto and Rinaldi, 2015). Direct observation of parents and children (by an objective third person) offers an alternative means of capturing this information and is particularly meaningful when conducted in naturalistic settings such as the home environment (Gardner, 2000; Wysocki, 2014). Several scoring frameworks have been developed for use with infants and toddlers (e.g., Indicator of Parent-Child Interaction, Dyadic Parent-Child Interaction Coding System), however, we identified a lack of suitable measures for 7–8-year-olds that could be feasibly used by trained non-clinician/student raters within the context of a large-scale study. Parenting (and parent-child interaction) naturally vary across ages and stages (Boldt et al., 2016), therefore it is critical to ensure that scoring frameworks are age- and developmentally appropriate.

In this paper, we draw on a large cohort of families participating in the school-age follow-up of a randomized controlled trial, to adapt an existing, validated measure of parent-child interaction previously used with 5–6-year-old children: the *Coding of Attached-Related Parenting (CARP)* Matias (2006). Specifically, this paper aims to:

(i) describe a protocol for adapting an existing scoring framework for measuring directly observed parent-child interaction (i.e., parents’ sensitive responding, parent-child positive mutuality) of 7–8-year-olds and their parents;
(ii) determine how parents’ sensitive responding and parent-child positive mutuality differ across the sample as a function of family demographic factors;
(iii) evaluate the psychometric properties of the newly developed measure (i.e., inter-rater reliability, construct validity).

## Materials and Methods

### Participant Recruitment

A total of 1,226 parents and their toddlers (aged 12–36 months) participated in the Early Home Learning Study (EHLS), a randomized controlled trial conducted in the Australian state of Victoria, between 2010 and 2013. Families of toddlers (aged 1–3 years) were recruited from ten metropolitan and regional local government areas. The Early Home Learning Study (EHLS) aimed to evaluate an early childhood parenting program called smalltalk, designed to support families to provide their young children with a stimulating home learning environment, to promote language and literacy development and school readiness (Nicholson et al., 2016; Hackworth et al., 2017). Families were recruited based on risk factors for poor child outcomes (i.e., low family income, receipt of government benefits, single parent, socially isolated or young parent ≤ 25 years, and culturally and linguistically diverse background). Parents were required to have sufficient oral English language skills to participate in the study (e.g., to take part in playgroups, complete parent questionnaires). Parents were ineligible if they were < 18 years old, did not speak English, were involved with child protection services, already received in-home support, or were deemed to require more intensive support services.

The smalltalk program was co-designed with early childhood professionals and parents, based on robust empirical evidence regarding the parenting behaviors known to facilitate children’s language development and school readiness (see Nicholson et al., 2016 for further details). The program focused on increasing the frequency with which parents practice the “five daily essentials”: (i) being warm and gentle, (ii) listening and talking more, (iii) tuning into their child, (iv) following their child’s lead, and (v) using everyday moments to teach their child something new. During the Early Home Learning Study (EHLS), 58 localities across the ten local government areas were randomly allocated to provide one of three study conditions to parents residing in pre-specified geographical boundaries: (i) a usual care supported (facilitated) playgroup, (ii) a smalltalk playgroup; or (iii) a smalltalk playgroup plus additional home coaching.

Approximately 5 years later, eligible families (*n* = 990) were invited to participate in EHLS at School Study to evaluate the longer-term impacts of smalltalk at child age 7.5 years (see Westrupp et al., 2018). Families were ineligible if they had actively withdrawn from the original study or had declined to be contacted regarding future research.

### Data Collection

Trained research assistants collected data via a home visit at child age 7.5 years between March 2016 and September 2018, including a parent-child observation and a parent questionnaire. Ethical approval was provided by La Trobe University Human Research Ethics Committee (No. 15-028).

#### Parent-Child Observation

Research assistants provided the parent and child with three toy sets: (i) LEGO^®^ Classic Box; (ii) Classic Jenga^®^ building blocks; and (iii) a set of animal ‘snap’ cards and asked them to play for “around 10 minutes.” They could use the toys “however they wanted” and could swap toys during the observation. If dyads swapped toy sets, they were asked to place the toys to the side and pack up at the end. The session was video-recorded using an Apple iPad Air^®^ fitted with a standing case. Where possible, the iPad^®^ was positioned on furniture (e.g., coffee table, arm of sofa) to minimize researcher-dyad eye contact. This method reduces the potential for observer reactivity and therefore enhances validity of the collected data (Bennetts et al., 2017a).

#### Parent Questionnaire

Parents were asked to complete a questionnaire on an iPad Air^®^ using REDCap, a secure data management platform (Research Electronic Data Capture Harris et al., 2009). The survey included demographic items and measures of parental health and wellbeing, parenting behaviors, and children’s behavior and development.

### Coding of Attachment-Related Parenting (CARP)

#### Original *CARP* Measure for Children Aged 5–6 Years

Informed by attachment and social learning theories, the *CARP* is a scoring framework designed to capture six domains of parent-child interaction: (i) parent sensitive responding, (ii) parent positive affect, (iii) parent negative affect, (iv) child positive affect, (v) child negative affect, and (vii) parent-child positive mutuality (Matias, 2006). According to the *CARP*, sensitive responding is defined as responsiveness that emphasizes the parent’s awareness of their child’s needs and sensitivity to their signals. Positive mutuality is defined as the quality of the interaction between parent and child, seeing both as a unique feature of the relationship. Validation data were collected from an at-risk community sample of 86 parent-child dyads in London, United Kingdom (child age 5–6 years). Dyads were video-recorded participating in three tasks: free play, LEGO^®^, and tidy-up. Observations were subsequently rated on a global scale from 1 to 7 for each of the six domains, for each of the three tasks (see Matias, 2006 for full scoring framework).

#### SCARP:7–8 Years for the EHLS at School Study

Adaptation of the *CARP* scoring framework was undertaken by authors SB and JL (referred to here as ‘master raters,’ due to their direct role in leading the adaptation), with support from authors JN and PL. Initial piloting of the original *CARP* using videos collected for the current study revealed three primary areas for refinement: (i) more tailored alignment of behaviors with those targeted by the smalltalk intervention; (ii) more age- and developmentally appropriate operationalization of the behaviors; and (iii) streamlining of the scales and rating procedures for reliable use by non-clinician/student raters. The adaptation process was iterative, characterized by regular testing, evaluation, re-testing, re-evaluation and team discussion. The primary adaptations are outlined in Table 1 and described below. We refer to this newly developed measure as the *SCARP:7–8 Years* or *SCARP* (i.e., short *CARP* for 7–8-year-old children).

**TABLE 1:** Summary of primary adaptations: *CARP* vs. *SCARP: 7–8 Years*.

Original *CARP*	*SCARP: 7–8 Years*
• 6 domains: child positive affect; child negative affect; parent positive affect; parent negative affect; sensitive responding; positive mutuality.	• 2 domains: sensitive responding; positive mutuality.
• Rating scale from 1 to 7.	• Rating scale from 1 to 5.
• Global rating of each domain (no specific element ratings).	• Individual assessment of 11 elements (yes/no/no opportunity) which informs selection of the domain score.
• Developed for children aged 5–6 years.	• Adapted for children aged 7–8 years.
• Free play, LEGO^®^, Tidy-up.	• Free play only.

Following careful piloting, we selected two of the original six domains for inclusion in the *SCARP* (sensitive responding, positive mutuality). There were several reasons for this decision. First, we wanted a direct measure of parents’ responsiveness and parent-child mutual responsiveness because there is robust evidence that these constructs are the most central aspects of attachment-related parenting and most predictive of children’s later outcomes (e.g., Moss et al., 1998; Ensor et al., 2012). Second, the behaviors rated for these two domains were most closely aligned with the smalltalk intervention (e.g., being warm and gentle, following the child’s lead). Third, some behaviors (e.g., negative affect) occur less frequently are therefore more difficult to observe (Gardner, 2000). This is especially the case with non-clinical populations for children of this age, as children are increasingly able to regulate their own behavior; thus, the resulting data would produce insufficient variability to be meaningful. Initial piloting also determined that reliably identifying and distinguishing between positive, neutral and negative affect (as per the original CARP) was problematic. Lastly, focusing on these two most crucial domains allowed us to create a briefer measure, to maximize rating efficiency and to support reliable use of the measure by non-clinicians within a large-scale research study. However, other researchers may wish to consider whether affect should be coded, or the use of specific toys or activities to elicit greater variability.

While the original *CARP* included separate ratings for free play and tidy-up components, piloting of the current study videos revealed that tidy-up was often very short (<1 min) with little meaningful variation within the cohort. Given that our study children were older than the validation sample, it is likely that they had greater capacity for self-regulation and required minimal parental support to conclude the free-play activity. The tidy-up activity was therefore not rated.

Agreed criteria for each element are shown in Table 2 (see Supplementary Files for further details, a copy of the full Manual is available upon request). While these elements are closely aligned with the original *CARP*, some modifications were required for 7–8-year-old children and to support reliable and efficient rating. For example, there were difficulties identifying how much shared attention/eye-contact and positive affect matching was ‘enough.’ As a result, we specified a frequency criterion for these two elements (i.e., at least three moments of eye-contact, at least three clear examples of simultaneous positive affect). This decision was made based on careful piloting of videos, during which we considered the typical frequency of these observed behaviors during the 5-minute videos and the number of instances that would be considered a fair and reasonable demonstration of these elements. We engaged in regular, in-depth conversations with the broader research team, many of whom have considerable expertise in the measurement of parent-child interaction and the development of rating frameworks (i.e., PL, JN, and SB).

**TABLE 2:** Operationalization of the 11 elements for the *SCARP: 7–8 Years*.

Sensitive responding	Positive mutuality
(1) *Responds to Requests for Help:* Child provides a clear verbal or non-verbal request for the parent to help and the parent provides an appropriate/timely response (note: ‘no opportunity’ if child does not request help). (2) *Responsive Engagement:* Parent is consistently engaged and responsive and follows the child’s lead during play. (3) *Facilitation/Guidance:* Parent makes reasonable efforts to offer guidance and facilitation during play (i.e., comments, suggestions, prompts or gestures that help to continue play and move the play forward). (4) *Encourages/Promotes Autonomy:* Parent makes reasonable efforts to promote the child’s autonomy through encouragement and praise (e.g., praise if child is doing well, or encouragement if child is struggling). (5) *Warmth:* Parent shows clear warmth toward the child throughout play (e.g., smiles, laughs, affectionate gestures).	(6) *Child Happily Involves Parent:* Child is clearly happy to be involving the parent throughout play. (7) *Interaction/Turn-taking:* There is clear evidence of consistent turn-taking OR interaction (working together to achieve the same goal). (8) *Shared Attention/Eye-contact:* Parent/child maintain attention on the same activity and each other throughout play AND this is supported by 3+ moments of eye contact. (9) *Positive Affect Matching:* Parent/child show 3+ clear examples of simultaneous positive affect (e.g., child giggles, parent laughs). (10) *Fluid Conversation:* Parent/child make reasonable efforts to talk about the activity throughout play. (11) *Shared Body Orientation:* Parent and child are both orientated toward one another (as much as possible, given disability or space constraints or needing to reach toys).

Other behaviors in the original *CARP* (see Matias, 2006) were removed if they were developmentally inappropriate for the current sample, observed too infrequently, or found to be too difficult to reliably identify. This process was important to assess whether assumptions made in observing dyads with younger children (i.e., 5–6-year-olds) hold for older children (7–8-year-old children), because the way in which parents respond to children as they grow older is likely to change to accommodate developmental differences (Gutman and Feinstein, 2010) (e.g., encouraging/assuming independence, using more complex language in communication, giving multiple instructions at once). For example, for parents’ sensitive responding, we removed ‘responding to ‘child’s non-verbal help-seeking behavior’ and responding to ‘child’s lost needing-behavior’ because piloting found these behaviors to be uncommon and difficult to reliably identify in this age group. We also removed ‘mirroring/matching’ from the positive mutuality domain, which relates to the parent and child matching or imitating each other’s behaviors or verbalizations. We found this to be rarely demonstrated in this age group and ostensibly occurs more often with younger children.

**FIGURE 1:**
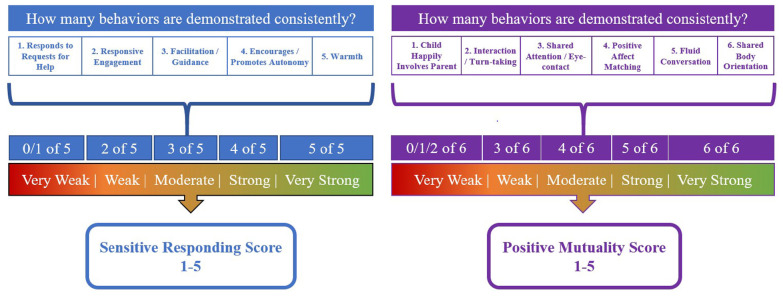
Overview of scoring process for the *SCARP: 7–8 Years.*

Early difficulties applying the 7-point scale reliably prompted evaluation of the original global rating scale for each domain. While the original 7-point scale would produce greater variability, we agreed that a 5-point scale would permit stronger inter-rater reliability. A two-step approach was proposed and tested, summarized in Figure 1 (see Supplementary Files for full rating sheet). Firstly, each of the 11 elements were individually assessed for consistency during the observation (yes/no rating), including the five sensitive responding elements and the six positive mutuality elements. For each domain, based on the number of elements demonstrated consistently, an overall domain score was selected (i.e., 1 = very weak; 2 = weak; 3 = moderate; 4 = strong; 5 = very strong).

### Staff Training and Monitoring

A suite of resources was developed for training purposes and to support a consistent understanding and application of the scoring framework. These included a comprehensive training manual with study-specific examples, a 1-page laminated scoring guide, and a detailed scoring and data entry protocol (see Supplementary Files, full manual available upon request). Fourteen master training videos were developed by the master raters using study video files selected to represent a diverse range of parent-child interactions. These 14 videos were independently rated by the master raters, discussed and finalized, then recorded on detailed scoring sheets for training purposes. The master raters were not clinicians, but both have a psychology background with experience conducting parenting research, including the assessment of parent behaviors and parent-child interactions (SB has a Ph.D. and JL has a bachelor with honors degree).

Four university students (3rd-year undergraduate and above) were trained to use the *SCARP* in January 2019. All students were completing relevant courses in education (primary, secondary or higher). Training included a 1-day workshop featuring video segments to illustrate the key behaviors and interactions, as well as an individual certification process. Certification required all students to rate at least three master videos until they achieved an average minimum of 80% agreement with master ratings. This is generally considered an appropriate and feasible benchmark for observational frameworks (e.g., Indicator of Parent-Child Interaction: Baggett et al., 2010). Master raters provided tailored feedback and support to students in between each video. Students achieved certification after five, six and eleven videos, respectively. A fourth student was provided with additional post-training support but was ultimately unable to achieve certification and withdrew.

A total of five raters (three students, two master raters) completed video scoring over a 10-week period, between January and March 2019. Raters processed around ten videos each per working day (approximately 20–25 minutes per video), interspersed with supplementary research or administrative activities to mitigate rating fatigue. Raters completed ‘refresher training’ at the start of every second shift, which involved rating another master video, followed by feedback from a master rater. This process helped to prevent ‘rating drift’ and to identify further training needs. Weekly group supervision meetings were held, to share progress and to discuss any questions raised.

### Scoring Protocol

Videos were pre-screened by the lead author (SB) to identify potential scoring issues (e.g., poor-quality lighting or audio, sibling interruptions, possible non-English language spoken, possible child disability) and these were allocated to SB and JL for rating. The team also discussed and double-scored any particularly difficult videos (e.g., videos with siblings frequently interrupting play, videos with difficult camera angles). An online Google Sheet^©^ was used to record scoring progress, in which raters were allocated their videos, updated the spreadsheet and noted any difficulties.

Videos were rated on a 5-minute segment, from minute 2:00 to minute 7:00. Commencing scoring at minute 2:00 allowed the dyad time to ‘warm up’ (Smith et al., 2019). Many dyads swapped toy sets during this period, so duration with each toy was recorded to the nearest 30 seconds, and ‘main toy’ was recorded as the toy set used by the child for the most time during the 5-minute segment. A small number of videos (*n* = 21, 3.5%) required a ‘time shift’ in the scoring period due to significant interruptions, parent/child leaving the play space, poor camera angle/audio or the initial use of a non-English language (before swapping to English at the research assistant’s request).

Scoring was undertaken using a hard copy scoring sheet, with space for notetaking and checkboxes to indicate both element and domain ratings. Data were immediately entered into REDCap. All scoring sheets were cross-checked with REDCap for clarity and accuracy of data entry by a second rater.

Every 10th video (*n* = 60) was independently double-rated. Inter-rater reliability was evaluated at 12 time points in blocks of five videos, to provide a measure of rating fidelity across the 10-week period. All five raters contributed to both the initial and double-rating process. This allowed for consistent monitoring of potential rating drift throughout the scoring period, and generated data to inform the focus of weekly supervision meetings.

### Parent-Reported Measures

Nine demographic variables known to be associated with parent-child interaction were examined: parent/child age (years), parent/child gender (1 = female, 2 = male); single parent status (0 = coupled, 1 = single); parent education (0 = year 12 and above, 1 = less than year 12); non-English speaking background (measured at baseline, 0 = no, 1 = yes); household unemployment (0 = both or one parent employed, 1 = both or single parent unemployed), and neighborhood disadvantage (SEIFA, where the Australian mean = 1000, sd = 100, lower scores indicate greater neighborhood disadvantage) (Australian Bureau of Statistics, 2018).

Six parent psychosocial and parenting measures were also used to examine construct validity of the *SCARP*. These parent-reported measures were not designed to precisely capture the same constructs as the *SCARP*, but were expected to be theoretically similar, or related, guided by the extant literature. We expected that associations between parent-reported and directly observed measures would be weak but in the expected direction, consistent with previous research (e.g., Bennetts et al., 2016; Bird et al., 2016). Thus, we anticipated that sensitive responding and positive mutuality would be positively associated with similar parent-reported constructs: warmth, consistency, self-efficacy and home activities with child. We predicted that sensitive responding and positive mutuality would be negatively correlated with parenting irritability and parent psychological distress, in-line with evidence that parents’ psychological state can impede parent-child interactions (Priel et al., 2019).

All parent-reported measures were commonly used and well-validated tools, used in large-scale national studies such as the Longitudinal Study of Australian Children (LSAC). The “home activities with child” measure modified for LSAC from the Early Childhood Longitudinal Study (National Center for Education Statistics, 2002) asks about the frequency of five home activities (e.g., “tell stories to your child”). Parent psychological distress (K6) is a commonly used and well-validated measure of psychological distress comprising 6 items on a 5-point scale (e.g., “nervous”) (Kessler et al., 2002). Parenting measures developed for the Longitudinal Study of Australian Children (LSAC) were administered on a 5-point scale, capturing parenting warmth (6 items, e.g., “Hug or hold your child for no particular reason”), parenting irritability (5 items, e.g., “How often are you angry when you punish this child?”), parenting self-efficacy (4 items, e.g., “Do you feel that this child’s behavior is more than you can handle?”) and parenting consistency (6 items, e.g., “When you give this child an instruction or make a request to do something, how often do you make sure that he/she does it?”) (Zubrick et al., 2014). Internal consistency of these measures for the current sample: psychological distress (α = 0.76); warmth (α = 0.83), irritability (α = 0.63), consistency (α = 0.64), home activities (α = 0.66), and parenting self-efficacy (α = 0.76).

### Statistical Analyses

Statistical analyses were conducted using Stata SE Version 14 (Statacorp, 2015). Intraclass correlation coefficients were used to evaluate inter-rater agreement for each domain (two-way mixed effects model to assess consistency of agreement, based on average ratings made on the same target). Percentage agreement was calculated to evaluate inter-rater reliability for each of the 11 elements. Differences in domain scores by toy choice were analyzed using Kruskal–Wallis test for categorical variables, with *post hoc* Dunn’s pairwise comparison. Multivariate linear regression analyses were used to examine differences in domain scores by family demographics, adjusted for toy choice (1 = LEGO^®^; Jenga^®^ = 2; Cards = 3), as well as three variables related to the original study, approximately 5 years earlier: condition allocation, local government area (recruitment site), and locality (physical site of playgroup attended).

## Results

### Participants

A total of 669 families participated in the EHLS at School Study (67.6% retention rate approximately 5 years after initial recruitment). Observations were collected from 601 parent-child dyads, of which five videos (0.8%) were not scorable (one corrupted file, one recorded in a non-English language, two with persistent sibling disruptions, and one with overly dark footage). A further four videos were rated for one of the two domains (one video could not be rated for positive mutuality, and three videos could not be rated for sensitive responding) due to poor audio, sibling disruption, use of a non-English language, and/or severe child disability. One video was recorded in a non-English language but could be scored due to one of the raters being fluent in that language.

For the included sample of 596 dyads, half used LEGO^®^ for most of the play session (51.5%), followed by Jenga^®^ (38.8%) and Cards (9.7%). Child age at assessment ranged from 7.0 to 8.6 years (mean = 7.5, sd = 0.3) and 49.8% were female. Parent age at assessment ranged from 25.0 to 65.4 years (mean = 39.7, sd = 5.4), most of whom were the child’s mother (96.0%), with a small number identifying as the child’s father, step-parent, extended family or other family caregiver.

Although families were initially recruited based on risk factors for poorer child outcomes, selective attrition resulted in the current retained sample being of relatively average socio-economic status (Bennetts et al., 2020). Less than one-fifth were single parents (13.4%) or had low education (secondary or less: 16.4%). Around one-third of families spoke a non-English language at home (30.9%). One in ten households (9.9%) consisted of either two unemployed parents or a single unemployed parent. On the whole, families were slightly more disadvantaged than the Australian mean according to the Index of Relative Socioeconomic Disadvantage (Australian Bureau of Statistics, 2018) based on participant postcodes: mean = 982.4, sd = 61.0, where the Australian *mean* is 1000 and sd is 100, and lower scores indicate greater neighborhood disadvantage. Most parents reported only low levels of psychological distress according to the Kessler-6 (mean = 4.0 out of 22, sd = 3.2) and most reported strong parenting self-efficacy (mean = 4.0 out of 5.0, sd = 0.6). Demographic and psychosocial variables were collected at the time of participation in the current study, except for language status, which was collected at initial recruitment to the Early Home Learning Study (EHLS).

### SCARP Scores

Frequencies for the 11 elements are presented in Table 3. Only six parents (1.0%) failed to respond appropriately to their child’s request for help. Due to insufficient variability, this element was not included in further analysis.

**TABLE 3:** Frequency of element ratings for sensitive responding and positive mutuality (*n* = 596)*.

Sensitive responding	Yes, *n* (%)	Positive mutuality	Yes, *n* (%)
Responds to request for help*	205 (34.5)	Child happily involves parent	519 (87.1)
Responsive engagement	487 (82.0)	Interaction/turn-taking	466 (78.2)
Facilitation/guidance	531 (89.4)	Shared attention/eye-contact	159 (26.7)
Encourages/promotes autonomy	212 (35.8)	Positive affect matching	222 (37.3)
Warmth	251 (42.3)	Fluid conversation	344 (57.7)
		Shared body orientation	536 (89.9)

*Responds to requests for help: No Opportunity (*n* = 383, 64.5%); No (*n* = 6, 1.0%). 1 video could only be assessed for sensitive responding and 3 videos could only be assessed for positive mutuality for reasons outlined above.

**FIGURE 2:**
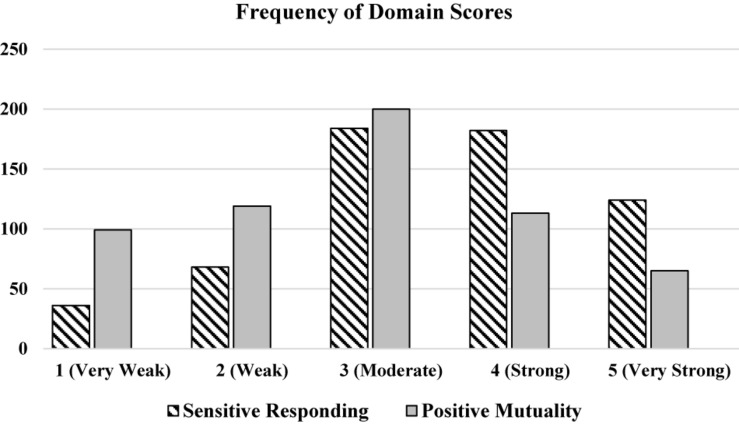
Frequency of domain scores for Sensitive Responding and Positive Mutuality (*n* = 596).

Both domains demonstrated good variability (sensitive responding: mean = 3.49, sd = 1.12; positive mutuality: mean = 2.88, sd = 1.22), although sensitive responding was negatively skewed and positive mutuality was normally distributed (Figure 2). A moderate, positive association was found between domain scores, indicating that they are related but distinct constructs (*r* = 0.49, *p* < 0.001).

### Inter-Rater Reliability

**FIGURE 3:**
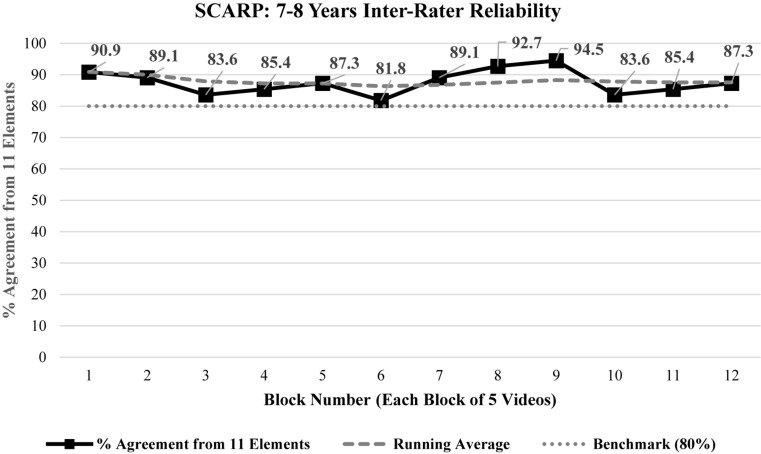
Inter-rater reliability for the 11 elements (calculated for each block of five videos).

Average agreement across the rating period was 87.6% (lowest block: 81.8%; highest block: 94.5%) (Figure 3). Average inter-rater agreement was consistently above the agreed benchmark of 80%. Percentage agreement for individual elements ranged from 71.7 to 96.7% (responsive engagement 85.0%; facilitation/guidance: 93.3%; encourages/promotes autonomy: 85.0%; warmth: 76.7%; child involving parent: 88.3%; interaction/turn-taking 96.7%; shared attention and eye contact: 96.7%; positive affect matching: 93.3%; fluid conversation 71.7%; shared body orientation 91.7%). Average intra-class correlation coefficients (ICCs) for the domains were 0.77 for sensitive responding and 0.84 for positive mutuality.

### Toy Choice

Differences in parent-child interaction by toy choice were observed during scoring, prompting further examination. Scores for both sensitive responding and positive mutuality varied significantly according to main toy used (*p* < 0.001 for both). For sensitive responding, the highest mean score was obtained for dyads who primarily used Cards (mean = 3.9, sd = 1.1) followed by Jenga^®^ (mean = 3.7, sd = 1.1) and LEGO^®^ (mean = 3.3, sd = 1.1). This pattern was consistent for positive mutuality, with the highest mean scores for Cards (mean = 3.7, sd = 1.2), followed by Jenga^®^ (mean = 3.3, sd = 1.2) and LEGO^®^ (mean = 2.4, sd = 1.0). Due to skewness of positive mutuality, we subsequently used the Kruskal–Wallis non-parametric test, which revealed that these were highly significant differences (sensitive responding: χ^2^ (2) = 23.23, *p* < 0.001; positive mutuality: χ^2^ (2) = 102.44, *p* < 0.001). Post-hoc pairwise tests indicated that, for both domains, mean scores for Jenga were significantly higher than Lego (*p* < 0.001) and mean scores for Cards were significantly higher than Lego (*p* < 0.001). The mean positive mutuality score for Cards was significantly higher than Jenga (*p* = 0.03) but this difference was not significant for sensitive responding (*p* = 0.09).

### Variations in SCARP Scores by Family Demographics

After adjusting for toy choice (as well as original study condition, local government area and locality), less sensitive responding was associated with non-English speaking background (*p* < 0.01), greater neighborhood disadvantage (*p* < 0.001), male parent (*p* = 0.04), and younger parent (*p* = 0.05). There was no significant difference in sensitive responding by child age (*p* = 0.82), child gender (*p* = 0.89), single parent (*p* = 0.39), parent education (*p* = 0.42), and household unemployment (*p* = 0.08).

Less positive mutuality was associated with male parent (*p* = 0.01), however there were no differences by non-English speaking background (*p* = 0.12), child age (*p* = 0.89), parent age (*p* = 0.12), child gender (*p* = 0.31), single parent (*p* = 0.59), parent education (*p* = 0.10), household unemployment (*p* = 0.10), and neighborhood disadvantage (*p* = 0.57).

Fully adjusted multivariate models are presented in Supplementary File 4, which explained 10% of the variance in sensitive responding and 21% of the variance in positive mutuality (adjusted *R*^2^ = 0.10 and 0.21, respectively). After full adjustment, fewer associations were significant: less sensitive responding remained associated with younger parent (*p* = 0.04), male parent (*p* = 0.03), non-English speaking background (*p* = 0.04), and greater neighborhood disadvantage (*p* = 0.02). None of the demographic variables were uniquely and significantly associated with positive mutuality.

### Construct Validity

Spearman’s correlation coefficients were calculated for all variables, given the presence of some skewness for sensitive responding, parenting self-efficacy, psychological distress and parenting warmth (Table 4). Overall, correlations between observed and parent-reported measures were negligible or weak to moderate, and were in the expected directions. Greater parent-reported irritability and psychological distress were generally associated with less observed sensitive responding and positive mutuality. Greater parent-reported parental warmth, consistency, self-efficacy and home activities were generally associated with greater observed sensitive responding and positive mutuality. The strongest correlation was between parent-reported psychological distress and ‘child happily involves parent’ (i.e., children were more likely to involve their parent in play when parents reported lower psychological distress).

**TABLE 4:** Associations between *SCARP: 7–8 Years* and parent-reported measures.

Directly observed	Parent-reported					
	Parenting warmth	Parenting irritability	Parenting consistency	Parenting self-efficacy	Home activities with child	Parent psychological distress
**Domains**						
Sensitive responding	0.09*	–0.05	0.10*	0.07	0.12**	–0.06
Positive mutuality	0.04	–0.06	0.05	0.06	0.07	−0.09*
**Elements**						
***Sensitive responding***						
Responsive engagement	0.08	–0.06	0.11**	0.12**	0.11**	−0.09*
Facilitation/guidance	0.02	–0.01	0.03	0.05	0.05	–0.08
Encourages/promotes autonomy	0.08*	–0.03	0.04	0.06	0.07	−0.08*
Warmth	0.05	–0.04	0.10*	–0.01	0.08*	0.02
***Positive mutuality***						
Child happily involves parent	0.04	–0.04	0.02	0.04	0.02	−0.16***
Interaction/turn-taking	0.01	0.00	–0.03	0.03	0.06	−0.10*
Shared attention/eye-contact	–0.01	–0.07	0.05	0.05	0.02	–0.07
Positive affect matching	–0.02	–0.06	0.05	0.03	0.01	–0.01
Fluid conversation	0.12**	0.01	0.06	0.01	0.11**	–0.02
Shared body orientation	0.03	–0.01	–0.01	0.00	0.02	–0.02

*p < 0.05, **p < 0.01, ***p < 0.001.

## Discussion

This paper describes a protocol for adapting a direct observational measure of parent-child interaction for 7–8-year-olds, to produce a measure that can be reliably used by non-clinicians. We draw on a large Australian community-based sample of parent-child dyads to offer methodological learnings, an examination of demographic differences, and an evaluation of inter-rater reliability and content validity. Findings suggest that the *SCARP* shows utility as a brief, direct measure of parent-child interaction for children aged 7–8 years, including parents’ sensitive responding and parent-child positive mutuality.

Firstly, this paper redresses a paucity of detailed protocols related to the development of direct observational parent-child interaction measures. Toy choice had a considerable impact on both domains, particularly positive mutuality. On average, dyads primarily using LEGO^®^ during the observation scored the lowest on both domains, and dyads using the Cards scored the highest. This may reflect the use of Cards and Jenga^®^ as inherently 2+ player games, compared to LEGO^®^ which may be associated with more independent play. As such, LEGO^®^ may have inhibited interaction, generating lower ratings. It is also possible that parents might struggle to actively engage with children if there are entrenched toy-based norms that emphasize solo play, as can be the case with LEGO^®^. Of note, LEGO^®^ was the main toy used by over half our dyads, highlighting its popularity as an almost universally recognized children’s toy. We recommend careful consideration and piloting of observational toys to ensure that they provide opportunity for turn-taking and interaction between parent and child. For example, more challenging games or puzzles might elicit more variability in parent-child interactions for this age group. These findings may have implications for practitioners working with families who are seeking to facilitate parent-child engagement and interaction. However, we also acknowledge that independent play is developmentally appropriate for 7–8-year-old children, such that a lack of positive mutuality in this context may not necessarily be problematic.

While inter-rater reliability was consistently high, warmth and fluid conversation had the lowest reliability between raters. This reflects existing evidence demonstrating weaker inter-rater reliability for more subjective behaviors and stronger inter-rater reliability for more observable or quantifiable behaviors (e.g., Brophy and Dunn, 2002). We recommend that careful attention be paid to these behaviors during training and throughout video rating, to support rating consistency. For example, one of our weekly supervision meetings focused on parental warmth, due to early difficulties consistently identifying this element. The group reviewed example video segments and discussed observable evidence of warmth. This process encouraged all raters to contribute to further refinement and operationalization of the elements. Associations with similar or related parent-reported measures provided evidence to support construct validity of the *SCARP*. Although associations were weak, they were in the expected directions, and align with previous evidence regarding the associations between directly observed and parent-reported measures (Bennetts et al., 2016). Of particular importance is the finding that children were more likely to involve parents in play when parents reported less psychological distress. This echoes previous evidence that parent mental health difficulties can impede quality parent-child interactions (e.g., Hakanen et al., 2019) and underscores the critical need for mental health support during the early years.

The withdrawal of one trainee before achieving certification highlights the complex nature of this work. Those learning to rate direct observations can experience a range of challenges, particularly if observed behaviors are brief or subtle. A supportive and encouraging team environment that accommodates varied learning styles is ideal, incorporating visual, written, group, and individual modes (Dantas and Cunha, 2020). Rigorous training and continued monitoring are critical, to ensure rating accuracy and to prevent rating drift. The current study involved five non-clinician raters (two research staff, three students), although further raters could be trained to more conclusively determine whether the framework can be reliably implemented by those without clinical training. For example, all three students were completing education degrees, although psychology courses typically cover measurement and psychometrics in much more detail. Regardless, we recommend that any group seeking to rate parent-child observations conducts careful training, cross-validation checks, and ongoing monitoring. We also acknowledge the inherent limitations of any measurement method; for example, direct observations can be subject to an ‘observer effect,’ such that participants may consciously or unconsciously adjust their behavior in the presence of the observer. We argue that both observational and parent-reported measures contribute uniquely to a more nuanced understanding of parent-child interaction (Gardner, 2000; Bennetts et al., 2016).

We also acknowledge challenges for administering and rating direct observations with families who speak a non-English language. This may be a particularly salient consideration given that one-third of our cohort reported speaking a non-English language at home. While speaking a non-English language was associated with less sensitive responding, it is certainly possible that parents would be able to demonstrate greater sensitive responding when using their home language (Bennetts et al., 2016, 2017a). This finding should therefore be interpreted with caution. Given that most rating frameworks are validated with English-speaking samples, there is a need for culturally and linguistically sensitive measures for observing parent-child interaction. Relatedly, cultural differences can shape parenting values and child-rearing goals, which in-turn influence parenting behaviors and children’s development. For example, Prevoo and Tamis-LeMonda (2017) reported cultural differences in parenting sensitivity, discipline style, child- vs. parent-led communications, and engagement in learning activities.

Given the time and resource intensive nature of direct observation, our sample is particularly large, generating robust evidence regarding parents’ sensitive responding and parent-child positive mutuality in the early school years. Further investigation is required to determine the utility and feasibility of this measure for use in practice settings, and to establish the predictive validity of the adapted *SCARP*, particularly for children’s socioemotional and behavioral development.

## Conclusion

In conclusion, this adapted measure of parent-child interaction for 7–8-year-olds offers rich insights into the parent-child relationship that cannot be captured via parent-report alone. We contribute here a detailed protocol for the adaptation and implementation process, including reflections about methodological learnings. The *SCARP:7–8 Years* addresses a gap in available parent-child interaction tools for use with community-based samples during the early school years and by non-clinician raters. Inter-reliability and content validity evidence suggest that the measure is psychometrically sound. The *SCARP* may prove useful for other research studies within this age group, or (subject to further validation) for clinicians working within a family-centered or attachment-based framework.

## Data Availability Statement

The datasets presented in this article are not readily available because ethical approval pertains to the use of collected data for the purposes of the current study. Any requests to access a deidentified dataset should be directed to the corresponding author but are subject to further ethical approval. Requests to access the datasets should be directed to ehlsatschool@latrobe.edu.au.

## Ethics Statement

Ethical approval for the EHLS at School Study was granted by the La Trobe University Human Research Ethics Committee (No. 15-028). Parents/caregivers provided written informed consent to participate in this study.

## Author Contributions

SB, JL, JN, PL, and EW conceived and designed the study. SB and JL performed the statistical analysis. SB wrote the first draft of the manuscript. JL, EW, NH, FM, JN, and PL all contributed to manuscript revision, read, and approved the submitted version. All authors contributed to the article and approved the submitted version.

## Conflict of Interest

The authors declare that the research was conducted in the absence of any commercial or financial relationships that could be construed as a potential conflict of interest.

## References

[B1] Aspland H. Gardner F. (2003). Observational measures of parent-child interaction: an introductory review. *Child Adoles. Ment. Health* 8 136–143. 10.1111/1475-3588.00061 32797579

[B2] Australian Bureau of Statistics (2018). *Socio-Economic Indexes for Areas (SEIFA) 2016 [Online].* Canberra: Australian Bureau of Statistics.

[B3] Baggett K. M. Carta J. J. Horn E. A. (2010). “The indicator of parent child interaction. Individual growth and developmental indicators: tools for monitoring progress and measuring growth in very young children,” in *Individual Growth and Developmental Indicators: Tools for Monitoring Progress and Measuring Growth in Very Young Children*, eds Carta J. Greenwood C. Walker D. Buzhardt J. (Baltimore, MD: Brookes).

[B4] Bardack S. Herbers J. E. Obradović J. (2017). Unique contributions of dynamic versus global measures of parent–child interaction quality in predicting school adjustment. *J. Fam. Psychol.* 31:649. 10.1037/fam0000296 28277709

[B5] Bennetts S. K. Love J. Hackworth N. J. Mensah F. K. Westrupp E. M. Berthelsen D. (2020). Selective attrition in longitudinal studies: effective processes for Facebook tracing. *Int. J. Soc. Res. Methodol.* 1–13. 10.1080/13645579.2020.1765104

[B6] Bennetts S. K. Mensah F. K. Westrupp E. M. Hackworth N. J. Reilly S. (2016). The agreement between parent-reported and directly measured child language and parenting behaviors. *Front. Psychol.* 7:1710. 10.3389/fpsyg.2016.01710 27891102 PMC5104739

[B7] Bennetts S. K. Mensah F. K. Green J. Hackworth N. J. Westrupp E. M. Reilly S. (2017a). Mothers’ experiences of parent-reported and video-recorded observational assessments. *J. Child Fam. Stud.* 26 3312–3326. 10.1007/s10826-017-0826-1

[B8] Bennetts S. K. Mensah F. K. Westrupp E. M. Hackworth N. J. Nicholson J. M. Reilly S. (2017b). Establishing agreement between parent-reported and directly-measured behaviours. *Australas. J. Early Child.* 42 105–115. 10.23965/AJEC.42.1.12

[B9] Bird A. L. Reese E. Taumoepea M. Schmidt J. Mohal J. Grant C. (2016). “You are our eyes and ears”: a new tool for observing parent-child interactions in large samples. *Long. Life Course Stud.* 7 386–408.

[B10] Bland J. M. Altman D. G. (1999). Measuring agreement in method comparison studies. *Stat. Methods Med. Res.* 8 135–160. 10.1191/096228099673819272 10501650

[B11] Boldt L. J. Kochanska G. Grekin R. Brock R. L. (2016). Attachment in middle childhood: predictors, correlates, and implications for adaptation. *Attach. Hum. Dev.* 18 115–140. 10.1080/14616734.2015.1120334 26673686 PMC4942850

[B12] Bornstein M. H. Tamis-LeMonda C. S. (1989). Maternal responsiveness and cognitive development in children. *New Direct. Child Adoles. Dev.* 1989 49–61. 10.1002/cd.23219894306 2710392

[B13] Bowlby J. (1997). *Attachment and Loss.* London: Pimlico.

[B14] Brophy M. Dunn J. (2002). What did mummy say? Dyadic interactions between young “hard to manage” children and their mothers. *J. Abnorm. Child Psychol.* 30 103–112. 12002392 10.1023/a:1014705314406

[B15] Criss M. M. Shaw D. S. Ingoldsby E. M. (2003). Mother–son positive synchrony in middle childhood: relation to antisocial behavior. *Soc. Dev.* 12 379–400. 10.1111/1467-9507.00239

[B16] Dantas L. A. Cunha A. (2020). An integrative debate on learning styles and the learning process. *Soc. Sci. Human. Open* 2:100017 10.1016/j.ssaho.2020.100017

[B17] Deater-Deckard K. Atzaba-Poria N. Pike A. (2004). Mother- and father-child mutuality in Anglo and Indian British families: a link with lower externalizing problems. *J. Abnorm. Child Psychol.* 32 609–620. 10.1023/b:jacp.0000047210.81880.14 15648528

[B18] Ensor R. Roman G. Hart M. J. Hughes C. (2012). Mothers’ depressive symptoms and low mother–toddler mutuality both predict children’s maladjustment. *Infant Child Dev.* 21 52–66. 10.1002/icd.762

[B19] Funamoto A. Rinaldi C. M. (2015). Measuring parent-child mutuality: a review of current observational coding systems. *Infant Ment. Health J.* 36 3–11. 10.1002/imhj.21481 25639997

[B20] Gardner F. (2000). Methodological issues in the direct observation of parent–child interaction: do observational findings reflect the natural behavior of participants? *Clin. Child Fam. Psychol. Rev.* 3 185–198. 11225753 10.1023/a:1009503409699

[B21] Gutman L. M. Feinstein L. (2010). Parenting behaviours and children’s development from infancy to early childhood: Changes, continuities and contributions. *Early Child Dev. Care* 180 535–556. 10.1080/03004430802113042

[B22] Hackworth N. J. Berthelsen D. Matthews J. Westrupp E. M. Cann W. Ukoumunne O. C. (2017). Impact of a brief group intervention to enhance parenting and the home learning environment for children aged 6–36 months: A cluster randomised controlled trial. *Prev. Sci.* 18 337–349. 10.1007/s11121-017-0753-9 28108927 PMC5352786

[B23] Hakanen H. Flykt M. Sinervä E. Nolvi S. Kataja E.-L. Pelto J. (2019). How maternal pre- and postnatal symptoms of depression and anxiety affect early mother-infant interaction? *J. Affect. Disord.* 257 83–90. 10.1016/j.jad.2019.06.048 31299408

[B24] Harris P. A. Taylor R. Thielke R. Payne J. Gonzalez N. Conde J. G. (2009). Research electronic data capture (REDCap)—a metadata-driven methodology and workflow process for providing translational research informatics support. *J. Biomed. Inform.* 42 377–381. 10.1016/j.jbi.2008.08.010 18929686 PMC2700030

[B25] Hedenbro M. Rydelius P.-A. (2019). Children’s abilities to communicate with both parents in infancy were related to their social competence at the age of 15. *Acta Paediatr.* 108 118–123. 10.1111/apa.14430 29869413

[B26] Iarocci G. Gardiner E. (2015). “Social competence during adolescence across cultures,” in *International Encyclopedia of the Social & Behavioral Sciences*, 2nd Edn, ed. Wright J. D. (Amsterdam: Elsevier), 216–221. 10.1016/b978-0-08-097086-8.23189-9

[B27] Keown L. J. (2012). Predictors of boys’ ADHD symptoms from early to middle childhood: the role of father–child and mother–child interactions. *J. Abnorm. Child Psychol.* 40 569–581. 10.1007/s10802-011-9586-3 22038253

[B28] Kessler R. C. Anderws G. Colpe L. J. Hiripi E. Mroczek D. K. Normand S.-L. T. (2002). Short screening scales to monitor population prevalences and trends in non-specific psychological distress. *Psychol. Med.* 32 959–976. 10.1017/s0033291702006074 12214795

[B29] Matias C. (2006). *Direct Observation of Parent-Child Interaction Based on Attachment Theory.* PhD Thesis London: Institute of Psychiatry, King’s College.

[B30] Morrison E. F. Rimm-Kauffman S. Pianta R. C. (2003). A longitudinal study of mother-child interactions at school entry and social and academic outcomes in middle school. *J. Schl. Psychol.* 41 185–200. 10.1016/s0022-4405(03)00044-x

[B31] Moss E. Rousseau D. Parent S. St-Laurent D. Saintonge J. (1998). Correlates of attachment at school age: maternal reported stress, mother-child interaction, and behavior problems. *Child Dev.* 69 1390–1405. 10.2307/1132273 9839423

[B32] National Center for Education Statistics (2002). *Early Childhood Longitudinal Study - Kindergarten Class of 1998-99 (ECLS-K) Psychometric Report for Kindergarten Through First Grade (NCES 2002-5).* Washington, DC: U.S Department of Education.

[B33] Nicholson J. M. Cann W. Matthews J. Berthelsen D. Ukoumunne O. C. Trajanovska M. (2016). Enhancing the early home learning environment through a brief group parenting intervention: study protocol for a cluster randomised controlled trial. *BMC Pediatr.* 16:73. 10.1186/s12887-016-0610-1 27255588 PMC4890293

[B34] Prevoo M. J. L. Tamis-LeMonda C. S. (2017). Parenting and globalization in western countries: explaining differences in parent–child interactions. *Curr. Opin. Psychol.* 15 33–39. 10.1016/j.copsyc.2017.02.003 28813265

[B35] Priel A. Zeev-Wolf M. Djalovski A. Feldman R. (2019). Maternal depression impairs child emotion understanding and executive functions: the role of dysregulated maternal care across the first decade of life. *Emotion* 20 1042–1058. 10.1037/emo0000614 31233315

[B36] Scherer E. Hagaman A. Chung E. Rahman A. O’donnell K. Maselko J. (2019). The relationship between responsive caregiving and child outcomes: evidence from direct observations of mother-child dyads in Pakistan. *BMC Public Health* 19:252. 10.1186/s12889-019-6571-1 30819173 PMC6396475

[B37] Smith J. Levickis P. Eadie T. Bretherton L. Conway L. Goldfeld S. (2019). Associations between early maternal behaviours and child language at 36 months in a cohort experiencing adversity. *Int. J. Lang. Commun. Disord.* 54 110–122. 10.1111/1460-6984.12435 30387273

[B38] Statacorp (2015). *Stata Statistical Software: Release 14.* College Station, TX: StataCorp LP.

[B39] Westrupp E. M. Bennett C. Cullinane M. Hackworth N. J. Berthelsen D. Reilly S. (2018). EHLS at School: school-age follow-up of the Early Home Learning Study cluster randomized controlled trial. *BMC Pediatr.* 18 148. 10.1186/s12887-018-1122-y 29720124 PMC5932854

[B40] Williams K. E. Berthelsen D. (2017). The development of prosocial behaviour in early childhood: contributions of early parenting and self-regulation. *Int. J. Early Child.* 49 73–94. 10.1007/s13158-017-0185-5

[B41] World Health Organization (2020). *Early Child Development [Online].* Geneva: World Health Organization.

[B42] Wysocki T. (2014). Introduction to the special issue: direct observation in pediatric psychology research. *J. Pediatr. Psychol.* 40 1–7. 10.1093/jpepsy/jsu104 25427552

[B43] Zubrick S. R. Lucas N. Westrupp E. M. Nicholson J. M. (2014). *Parenting Measures in the Longitudinal Study of Australian Children: Construct Validity and Measurement Quality, Waves 1 to 4.* Canberra: Commonwealth of Australia.

