# Supplementary figures and images for: Protocol for the Adaptation of a Direct Observational Measure of Parent-Child Interaction for Use With 7–8-Year-Old Children
Source: Front Psychol. 2021 Jan 14;11:619336. doi: 10.3389/fpsyg.2020.619336 (PMC7857049; doi:10.3389/fpsyg.2020.619336)

**Supplementary File 1.** *SCARP: 7-8 Years* Brief Scoring Guide


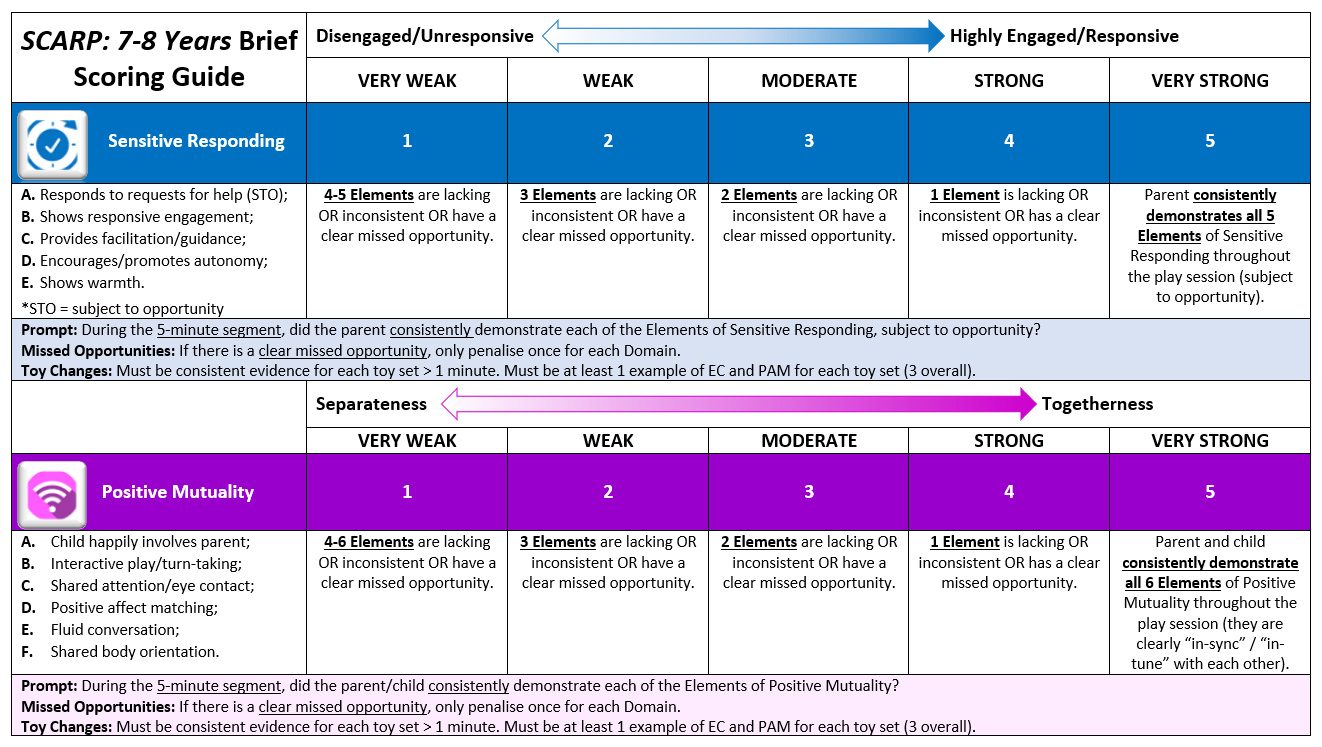

Supplement: Supplementary file 2 [file Table_1.docx]

**Supplementary File 2.** *SCARP:7-8 Years* Guide to Rating Each Element.


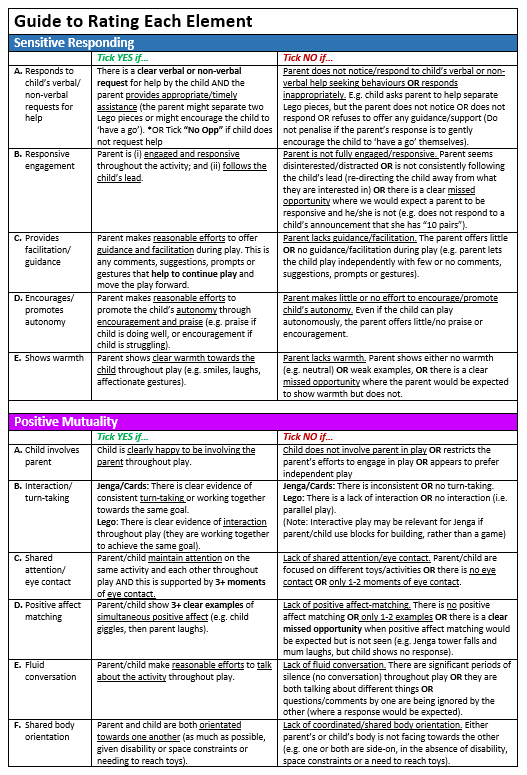

Supplement: Supplementary file 3 [file Table_2.docx]

**Supplementary File 3.** Scoring Sheet for *SCARP:7-8 Years*


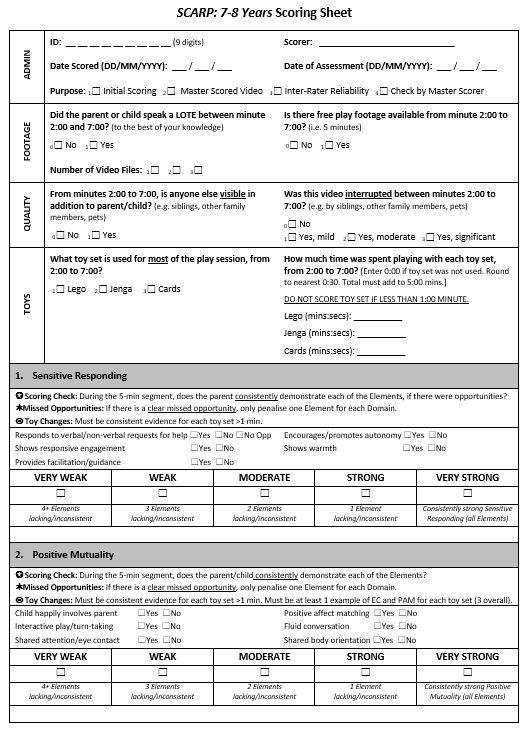

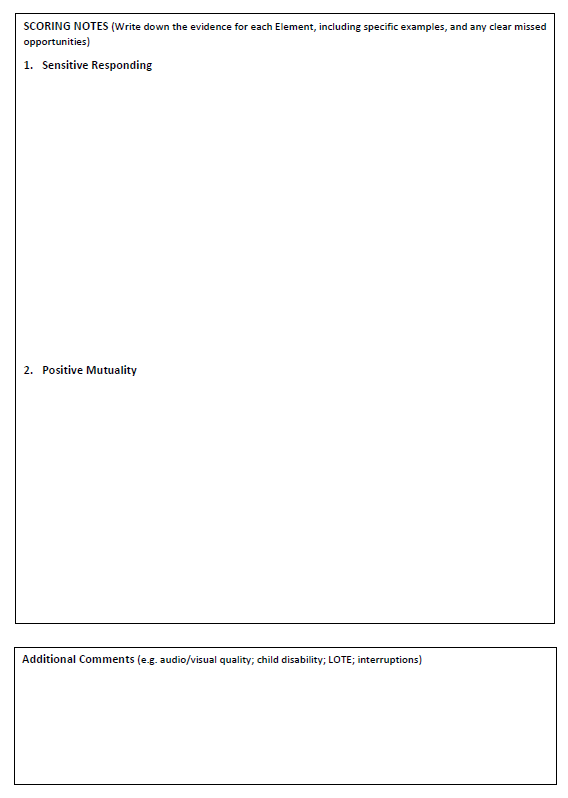

Supplement: Supplementary file 4 [file Table_3.docx]
