# Supplementary material for: Protocol for the Adaptation of a Direct Observational Measure of Parent-Child Interaction for Use With 7–8-Year-Old Children
Source: Front Psychol. 2021 Jan 14;11:619336. doi: 10.3389/fpsyg.2020.619336 (PMC7857049; doi:10.3389/fpsyg.2020.619336)
Supplement: Supplementary file 5 [file Table_4.docx]

**Supplementary File 4.** Adjusted associations between sensitive responding, positive mutuality and demographic factors.

|  | Sensitive responding | | Positive mutuality | |
| --- | --- | --- | --- | --- |
|  | Coefficient (95% CI) | *p* | Coefficient (95% CI) | *p* |
| Child age (years) | -.12 (-.45, .22) | .49 | -.03 (-.37, .31) | .86 |
| Parent age (years) | .02 (.00, .03) | .04 | -.01 (-.03, .01) | .30 |
| Child gender (male) | -.01 (-.19, .16) | .90 | -.11 (-.28, .07) | .23 |
| Parent gender (male) | -.57 (-1.08, -.07) | .03 | -.50 (-1.02, .01) | .05 |
| Language other than English | -.22 (-.43, -.01) | .04 | -.12 (-.33, .09) | .26 |
| Single parent | -.09 (-.38, .21) | .56 | .04 (-.26, .33) | .81 |
| Parent without year 12 | -.02 (-.38, .35) | .93 | -.24 (-.61, .13) | .21 |
| Household unemployment | -.16 (-.49, .17) | .35 | -.17 (-.50, .17) | .33 |
| Neighbourhood disadvantage | .002 (.000, .003) | .02 | .0002 (-.001, .002) | .78 |
|  |  |  |  |  |
|  | Adj R^2^ = .10 |  | Adj R^2^ = .21 |  |

*Adjusted for main toy used, allocated condition, local government area, and locality (during participation in RCT approximately five years earlier)
